# Benefits of using removable filters in dual-layer flat panel detectors

**DOI:** 10.1088/1361-6560/acc77d

**Published:** 2023-04-07

**Authors:** Emily Y Cai, Christian De Caro, Kevin Treb, Ke Li

**Affiliations:** 1 Department of Medical Physics, School of Medicine and Public Health, University of Wisconsin-Madison, 1111 Highland Avenue, Madison, WI 53705, United States of America; 2 Department of Radiology, School of Medicine and Public Health, University of Wisconsin-Madison, 600 Highland Avenue, Madison, WI 53792, United States of America

**Keywords:** dual-layer flat panel detector, dual-energy x-ray imaging, x-ray detector

## Abstract

*Objective.* Existing dual-layer flat panel detectors (DL-FPDs) use a thin scintillator layer to preferentially detect low-energy x-rays, followed by a permanent Cu filter to absorb residual low-energy x-rays, and finally, a thicker scintillator layer to preferentially detect high-energy x-rays. The image outputs of the two scintillator layers can be jointly processed for dual-energy (DE) planar and cone-beam CT imaging. In clinical practice, a given FPD is often used for not only DE imaging but also routine single-energy (SE) imaging. With the permanent Cu layer, the total x-ray absorption is unsatisfactory for SE imaging since more than 30% of x-rays can be lost in the Cu layer. The purpose of this work was to demonstrate the benefits of using a removable filter material in DL-FPDs for SE and DE imaging applications. *Approach.* The proposed detector contains a removable filter between the two scintillator layers. The filter can be either a chamber filled with a liquid high-*Z*_eff_ material or a removable solid filter. When DE imaging is not clinically indicated, the DL-FPD can switch to a high-efficiency SE imaging mode by retracting the filter from the inter-scintillator space. For commonly available filter materials (iodine, gadolinium, and Cu), their optimal area densities were theoretically calculated for both water-bone decomposition and water-iodine decomposition DE imaging tasks. Preliminary experimental studies were also performed to compare the SE performance of the proposed DL-FPD with the existing DL-FPD with the permanent Cu filter and study the stability of the liquid filter on a rotating gantry. *Main results.* The optimal filter material was found to be an iodine solution (approximately 375 mg cm^−2^). With this liquid filter in place, the proposed DL-FPD has equivalent or better DE imaging performance compared with the existing DL-FPD with the Cu filter. When the filter is removed from the inter-scintillator space for SE imaging, the total x-ray absorption efficiency of the proposed DL-FPD ranges from 73% (100 kVp) to 54% (140 kVp), compared with 51% (100 kVp) to 41% (140 kVp) for the existing DL-FPD with a permanent 1 mm Cu filter. *Significance.* The removable filter provides a boost to the total x-ray absorption efficiency of DL-FPDs for SE imaging without compromising DE imaging. This can facilitate the adoption of DL-FPDs in clinical x-ray imaging systems that usually perform more SE imaging procedures than DE imaging series.

## 1. Introduction

The concept of dual-layer x-ray detectors dates back to the 1980s when Barnes *et al* (1985) used a thin (86 *μ*m) layer of the yttrium-oxysulfide scintillator (Y_2_O_2_S, *Z*_eff_ = 35) to preferentially detect low-energy x-rays, a 0.38 mm Cu layer to preferentially filter out residual low-energy x-rays, and finally a 164 *μ*m layer of gadox scintillator (Gd_2_O_2_S, *Z*_eff_ = 58) to preferentially detect high-energy x-rays. The low- and high-energy x-ray images were combined linearly to obtain soft-tissue (bone cancellation) and bone (soft-tissue cancellation) radiographs. The authors noted that ‘the energy separation achieved with the dual-energy (DE) detector is markedly reduced when the copper filter is removed. In practice, this decrease overshadows the advantage of the increased high energy image detected fluence.’

**Figure 1.**
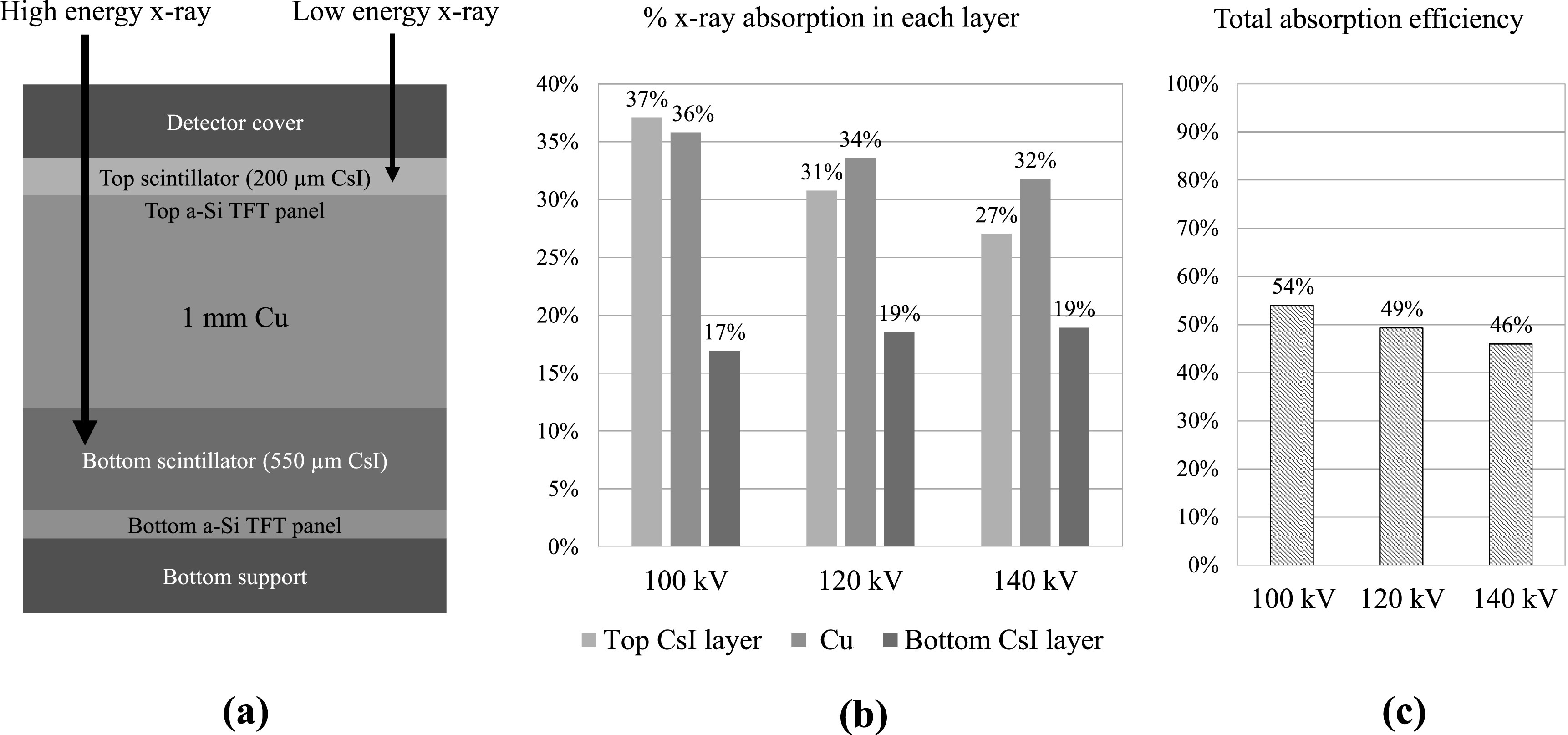
(a) A schematic of the cross-section of the existing DL-FPD. The thicknesses of the scintillator and Cu layers were drawn to scale. (b) Percentage of x-rays absorbed in each scintillator (CsI) layer and the Cu layer. (c) Total percentage of x-rays absorbed in the two scintillator layers when the Cu layer is present.

Based upon the pioneering work of Barnes *et al*, additional developments of dual-layer flat panel detectors (DL-FPDs) with digital readouts were recently reported (Lu *et al* 2019, 2021). As shown in figure 1(a), the two scintillator layers are made of the same material (e.g. CsI:Tl) but with different thicknesses (200 *μ*m versus 550 *μ*m). Compared with (Barnes *et al* 1985), the thickness of the Cu layer in recent DL-FPDs has been increased aggressively to 1 mm to widen the energy separation between the two CsI layers. Research has been done to explore DL-FPDs’ potential clinical applications, including DE radiography with bone suppression, soft-tissue motion tracking, mask-free digital subtraction angiography, metal artifact suppression in cone-beam CT (CBCT), material quantification and discrimination, scatter estimation, improved COVID-19 detection, etc (Lu *et al* 2019, Shi *et al* 2020, 2020, Ma *et al* 2020, Stahl *et al* 2021, Shi *et al* 2021, 2021, Wang *et al* 2021, Wang 2021). Compared to kVp-switching-based DE imaging methods, ‘the simultaneous low and high energy acquisition is an advantage, not only in reducing misregistration problems but also in reducing the x-ray tube load.’ (Barnes *et al* 1985) Compared with energy-discriminating photon-counting detectors (PCDs), DL-FPDs offer more extensive spatial coverage with a much lower system cost at the moment. For high-flux imaging procedures, DL-FPDs do not suffer from pileup-induced count losses and image artifacts as PCDs do.

Despite their advantages in DE imaging, existing digital DL-FPDs have a major technical limitation: as shown in figures 1(b)–(c), the Cu layer sandwiched between the two scintillator layers can absorb a significant fraction of the input x-rays (e.g. 34% at 120 kV). While this is justifiable for DE applications where a better energy separation may outweigh some photon loss, for other single-energy (SE) imaging applications, the loss of x-rays in the Cu layer is unnecessary and degrades the overall radiation dose efficiency of the detector system. For medical imaging systems that use FPDs, SE acquisitions are usually more common than DE acquisitions. Taking chest radiography as an example, as stated by clinical experts in a topic review: ‘DE subtraction is most helpful when a lung lesion is obscured by overlying bone structures. If a nodule is obscured by an overlapping soft-tissue structure, DE subtraction may not be beneficial.’ (Kuhlman *et al* 2006) Additionally, the majority of lateral-view chest radiographs are acquired with the SE mode due to a lack of low-energy photons. For image-guided radiation therapy, while FPDs may potentially benefit from DE imaging when tracking moving tumors such as those in the lung (Lu *et al* 2021), the same detectors are often used for simpler geometric alignment tasks where SE imaging is sufficient (Boda-Heggemann *et al* 2011, Posiewnik and Piotrowski 2019). For chest CT imaging, a recent survey conducted by the Society of Thoracic Radiology shows that among institutions with DE-CT scanners, only 1.9% used the DE mode for all patients scanned; for the overwhelming majority of the patients, DE was individually chosen for patients depending on the specific clinical indication (Rajiah 2020). For interventional x-ray systems with FPDs, the vast majority of imaging tasks are the localization of high-contrast devices and anatomy that do not require or benefit from DE imaging (Shalom *et al* 2020). For those SE imaging procedures with extended scan duration, concerns over potential radiation effects (Balter *et al* 2010) make the presence of a permanent Cu filter in the FPD highly undesirable due to the reduction in the SE imaging mode’s radiation absorption efficiency.

This work presents a new DL-FPD design with a removable filter layer between the two scintillator layers. Unlike the permanent Cu filter in existing DL-FPDs, the filter material can be removed from the inter-scintillator space when the DL-FPD is used for SE imaging procedures to avoid unnecessary x-ray loss. The following section describes theoretical and experimental methods to demonstrate the potential benefits of using removable filters in DL-FPDs.

## 2. Methods

### 2.1. Detector design

**Figure 2.**
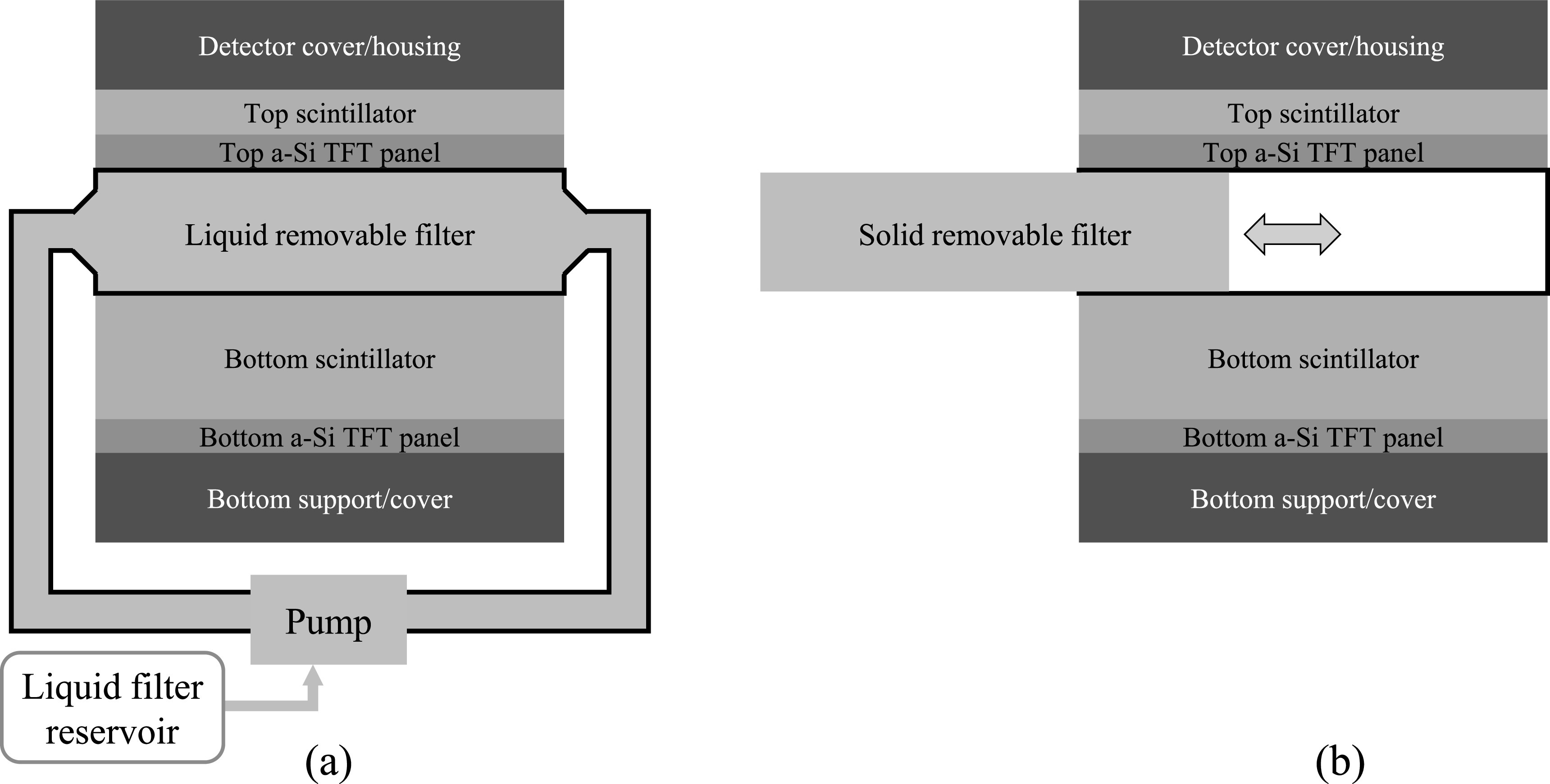
Two designs of removable filters for DL-FPDs. (a) A liquid filter device. Before SE imaging, the liquid material can be drained to a reservoir so that no photons are wasted in the filter. For DE imaging procedures, the liquid can be pumped back to the inter-scintillator space to improve spectral separation. (b) A solid removable filter device. An open space between the top and bottom scintillator can be created, such that a solid metallic filter can be inserted or removed depending on the imaging applications.

As shown in figure 2(a), the removable filter can be implemented using a watertight chamber between the two scintillator layers. Before DE imaging procedures, the chamber can be filled with liquid high-*Z*_eff_ materials such as iodine solutions. When DE imaging is not clinically indicated, the FPD can return to the high-efficiency SE imaging mode by draining the liquid filter from the inter-scintillator space to an off-detector reservoir using a pump. Alternatively, a solid removable filter can be manually inserted or removed from the inter-scintillator space in DL-FPD, as shown in 2(b).

This work studied three filter materials, including a gadolinium (Gd) liquid solution, an iodine (I) liquid solution, and a solid Cu removable filter. The goal was to determine the optimal area density, *s*_f_ = *ρ*_f_ *T*_f_, of the removable filter as a function of the input x-ray spectrum, DE imaging task, and thickness of the first CsI layer (*T*_1_). The second CsI layer’s thickness, *T*_2_, was kept at 550 *μ*m (the same as what’s in the existing DL-FPDs).

Assuming the DE imaging task is to estimate water and bone thicknesses via a two-material decomposition, we modeled the expected outputs of the two scintillator layers as follows:

\begin{eqnarray*}{\bar{I}}_{1}={\int }_{0}^{{E}_{{\mathrm{m}}}}[\bar{N}(E)\,E\,{{\mathrm{e}}}^{-{A}_{{\mathrm{w}}}{f}_{{\mathrm{w}}}(E)-{A}_{{\mathrm{b}}}{f}_{{\mathrm{b}}}(E)}]\left(1-{{\mathrm{e}}}^{-{\mu }_{\mathrm{CsI}}(E){T}_{1}}\right){\mathrm{d}}E;\end{eqnarray*}

\begin{eqnarray*}{\bar{I}}_{2}={\int }_{0}^{{E}_{{\mathrm{m}}}}[\bar{N}(E)\,E\,{{\mathrm{e}}}^{-{A}_{{\mathrm{w}}}{f}_{{\mathrm{w}}}(E)-{A}_{{\mathrm{b}}}{f}_{{\mathrm{b}}}(E)}]\,{{\mathrm{e}}}^{-{\mu }_{\mathrm{CsI}}(E){T}_{1}-{\mu }_{{\mathrm{f}}}(E){T}_{{\mathrm{f}}}}\left(1-{{\mathrm{e}}}^{-{\mu }_{\mathrm{CsI}}(E){T}_{2}}\right){\mathrm{d}}E.\end{eqnarray*}

In the above two equations, $\bar{N}$ is the expected number of input x-ray photons with energy *E*; *E*_m_ is the maximum x-ray energy; *f*_w_(*E*) and *f*_b_(*E*) denote the mass attenuation coefficients of water and bone, respectively; *A*_w_ and *A*_b_ are the projections of the patient attenuation into the water basis and bone basis, respectively; *μ*_f_ is the linear attenuation coefficient of the liquid filter material. Taking an iodine-water solution with an iodine concentration of *ρ*_*f*_ as an example, *μ*_f_ can be calculated as

\begin{eqnarray*}{\mu }_{{\mathrm{f}}}(E)=\displaystyle \frac{{\rho }_{{\mathrm{f}}}}{{\rho }_{{\mathrm{I}}}}\,{\mu }_{{\mathrm{I}}}(E)+\left(1-\displaystyle \frac{{\rho }_{{\mathrm{f}}}}{{\rho }_{{\mathrm{I}}}}\right)\,{\mu }_{{\mathrm{w}}}(E),\end{eqnarray*}

where *μ*_I_ and *μ*_w_ denote the attenuation coefficients of pure iodine and water, respectively, and *ρ*_I_ denotes the mass density of pure iodine.

For energy-integrating FPDs, the variances of the two scintillator layers’ outputs were modeled as (Swank 1973)

\begin{eqnarray*}{\sigma }_{{I}_{1}}^{2}={\int }_{0}^{{E}_{{\mathrm{m}}}}[\bar{N}(E){E}^{2}\,{{\mathrm{e}}}^{-{A}_{{\mathrm{w}}}{f}_{{\mathrm{w}}}(E)-{A}_{{\mathrm{b}}}{f}_{{\mathrm{b}}}(E)}]\,(1-{{\mathrm{e}}}^{-{\mu }_{\mathrm{CsI}}(E){T}_{1}}){\mathrm{d}}E;\end{eqnarray*}

\begin{eqnarray*}{\sigma }_{{I}_{2}}^{2}={\int }_{0}^{{E}_{{\mathrm{m}}}}[\bar{N}(E)\,{E}^{2}\,{{\mathrm{e}}}^{-{A}_{{\mathrm{w}}}{f}_{{\mathrm{w}}}(E)-{A}_{{\mathrm{b}}}{f}_{{\mathrm{b}}}(E)}]\,{{\mathrm{e}}}^{-{\mu }_{\mathrm{CsI}}(E){T}_{1}-{\mu }_{{\mathrm{f}}}(E){T}_{{\mathrm{f}}}}\left(1-{{\mathrm{e}}}^{-{\mu }_{\mathrm{CsI}}(E){T}_{2}}\right){\mathrm{d}}E.\end{eqnarray*}

In clinical practice, ${\bar{I}}_{1}$ and ${\bar{I}}_{2}$ are generally unavailable. Instead, only a single sample of each, *I*_1_ and *I*_2_, are acquired. An important goal of DE imaging is to estimate *A*_w_ and *A*_b_ of the patient from *I*_1_ and *I*_2_. Assuming *I*_1_ and *I*_2_ follow a normal distribution with the following probability density functions:

\begin{eqnarray*}{p}_{1}({I}_{1})=\displaystyle \frac{1}{{\left(2\pi {\sigma }_{{I}_{1}}^{2}\right)}^{1/2}}\,{{\mathrm{e}}}^{-\tfrac{{\left({I}_{1}-\bar{{I}_{1}}\right)}^{2}}{2{\sigma }_{{I}_{1}}^{2}}},\qquad {p}_{2}({I}_{2})=\displaystyle \frac{1}{{\left(2\pi {\sigma }_{{I}_{2}}^{2}\right)}^{1/2}}\,{{\mathrm{e}}}^{-\tfrac{{\left({I}_{2}-\bar{{I}_{2}}\right)}^{2}}{2{\sigma }_{{I}_{2}}^{2}}},\end{eqnarray*}

then for the two parameters-of-interest (*A*_w_ and *A*_b_), their joint log-likelihood function is given by:

\begin{eqnarray*}{ \mathcal L }({A}_{{\mathrm{w}}},{A}_{{\mathrm{b}}}| {I}_{1},{I}_{2})=-\mathrm{ln}{\sigma }_{{I}_{1}}-\mathrm{ln}{\sigma }_{{I}_{2}}-\displaystyle \frac{{\left({I}_{1}-\bar{{I}_{1}}\right)}^{2}}{2{\sigma }_{{I}_{1}}^{2}}-\displaystyle \frac{{\left({I}_{2}-\bar{{I}_{2}}\right)}^{2}}{2{\sigma }_{{I}_{2}}^{2}}-\mathrm{ln}(2\pi ).\end{eqnarray*}

As shown previously by Roessl and Herrmann (2009), the elements of the 2 × 2 Fisher information matrix (FIM) for *A*_w_ and *A*_b_ are given by:

\begin{eqnarray*}{{ \mathcal F }}_{11}=E\left[-\displaystyle \frac{{\partial }^{2}{ \mathcal L }}{{\partial }^{2}{A}_{{\mathrm{w}}}}\right]\end{eqnarray*}

\begin{eqnarray*}=\displaystyle \frac{1}{{\sigma }_{{I}_{1}}^{2}}{\left(\displaystyle \frac{\partial {\bar{I}}_{1}}{\partial {A}_{{\mathrm{w}}}}\right)}^{2}+\displaystyle \frac{1}{{\sigma }_{{I}_{2}}^{2}}{\left(\displaystyle \frac{\partial {\bar{I}}_{2}}{\partial {A}_{{\mathrm{w}}}}\right)}^{2}+\displaystyle \frac{1}{2{\left({\sigma }_{{I}_{1}}^{2}\right)}^{2}}{\left(\displaystyle \frac{\partial {\sigma }_{{I}_{1}}^{2}}{\partial {A}_{{\mathrm{w}}}}\right)}^{2}+\displaystyle \frac{1}{2{\left({\sigma }_{{I}_{2}}^{2}\right)}^{2}}{\left(\displaystyle \frac{\partial {\sigma }_{{I}_{2}}^{2}}{\partial {A}_{{\mathrm{w}}}}\right)}^{2};\end{eqnarray*}

\begin{eqnarray*}\begin{array}{rcl}{{ \mathcal F }}_{22} &amp; = &amp; E\left[-\displaystyle \frac{{\partial }^{2}{ \mathcal L }}{{\partial }^{2}{A}_{{\mathrm{b}}}}\right]\\ &amp; = &amp; \displaystyle \frac{1}{{\sigma }_{{I}_{1}}^{2}}{\left(\displaystyle \frac{\partial {\bar{I}}_{1}}{\partial {A}_{{\mathrm{b}}}}\right)}^{2}+\displaystyle \frac{1}{{\sigma }_{{I}_{2}}^{2}}{\left(\displaystyle \frac{\partial {\bar{I}}_{2}}{\partial {A}_{{\mathrm{b}}}}\right)}^{2}+\displaystyle \frac{1}{2{\left({\sigma }_{{I}_{1}}^{2}\right)}^{2}}{\left(\displaystyle \frac{\partial {\sigma }_{{I}_{1}}^{2}}{\partial {A}_{{\mathrm{b}}}}\right)}^{2}+\displaystyle \frac{1}{2{\left({\sigma }_{{I}_{2}}^{2}\right)}^{2}}{\left(\displaystyle \frac{\partial {\sigma }_{{I}_{2}}^{2}}{\partial {A}_{{\mathrm{b}}}}\right)}^{2};\end{array}\end{eqnarray*}

\begin{eqnarray*}\begin{array}{rcl}{{ \mathcal F }}_{12} &amp; = &amp; {{ \mathcal F }}_{21}=E\left[-\displaystyle \frac{{\partial }^{2}{ \mathcal L }}{\partial {A}_{{\mathrm{w}}}\partial {A}_{{\mathrm{b}}}}\right]\\ &amp; = &amp; \displaystyle \frac{1}{{\sigma }_{{I}_{1}}^{2}}\displaystyle \frac{\partial {\bar{I}}_{1}}{\partial {A}_{{\mathrm{w}}}}\displaystyle \frac{\partial {\bar{I}}_{1}}{\partial {A}_{{\mathrm{b}}}}+\displaystyle \frac{1}{{\sigma }_{{I}_{2}}^{2}}\displaystyle \frac{\partial {\bar{I}}_{2}}{\partial {A}_{{\mathrm{w}}}}\displaystyle \frac{\partial {\bar{I}}_{2}}{\partial {A}_{{\mathrm{b}}}}+\displaystyle \frac{1}{2{\left({\sigma }_{{I}_{1}}^{2}\right)}^{2}}\displaystyle \frac{\partial {\sigma }_{{I}_{1}}^{2}}{\partial {A}_{{\mathrm{w}}}}\displaystyle \frac{\partial {\sigma }_{{I}_{1}}^{2}}{\partial {A}_{{\mathrm{b}}}}+\displaystyle \frac{1}{{\left({\sigma }_{{I}_{2}}^{2}\right)}^{2}}\displaystyle \frac{\partial {\sigma }_{{I}_{2}}^{2}}{\partial {A}_{{\mathrm{w}}}}\displaystyle \frac{\partial {\sigma }_{{I}_{2}}^{2}}{\partial {A}_{{\mathrm{b}}}}.\end{array}\end{eqnarray*}

The partial derivatives in equations (8)–(11) can be calculated based on equations (1), (2), (4) and (5). For example

\begin{eqnarray*}\displaystyle \frac{\partial {\bar{I}}_{1}}{\partial {A}_{{\mathrm{w}}}}=-{\int }_{0}^{{E}_{{\mathrm{m}}}}[\bar{N}(E)\,E\,{f}_{{\mathrm{w}}}(E)\,{{\mathrm{e}}}^{-{A}_{{\mathrm{w}}}{f}_{{\mathrm{w}}}(E)-{A}_{{\mathrm{b}}}{f}_{{\mathrm{b}}}(E)}]\,(1-{{\mathrm{e}}}^{-{\mu }_{\mathrm{CsI}}(E){T}_{1}}){\mathrm{d}}E.\end{eqnarray*}

From the FIM, the Cramér–Rao lower bound (CRLB) of parameters *A*_w_ and *A*_b_ can be calculated:

\begin{eqnarray*}{\tilde{\sigma }}_{{A}_{{\mathrm{w}}}}^{2}=\displaystyle \frac{{{ \mathcal F }}_{22}}{| { \mathcal F }| };\end{eqnarray*}

\begin{eqnarray*}{\tilde{\sigma }}_{{A}_{{\mathrm{b}}}}^{2}=\displaystyle \frac{{{ \mathcal F }}_{11}}{| { \mathcal F }| }.\end{eqnarray*}

For the optimization of *T*_f_ and *T*_1_, two DE tasks were used. The first one is to estimate water and bone thicknesses with the following ground truth: *A*_w_ = 30 g cm^−2^; *A*_b_ = 10 g cm^−2^. The second is to estimate water and iodine thickness (via water-iodine 2-material decomposition) with the following ground truth: *A*_w_ = 30 g cm^−2^; *A*_I_ = 250 mg cm^−2^. *T*_2_ was fixed at 550 *μ*m and the input spectrum was a typical 120 kVp beam with 2.5 mm Al inherent filtration and 1 mm Cu external beam filter. For both DE tasks, CRLB was also evaluated for the existing DL-FPD with the permanent 1 mm Cu filter.

### 2.2. Experimental study

**Figure 3.**
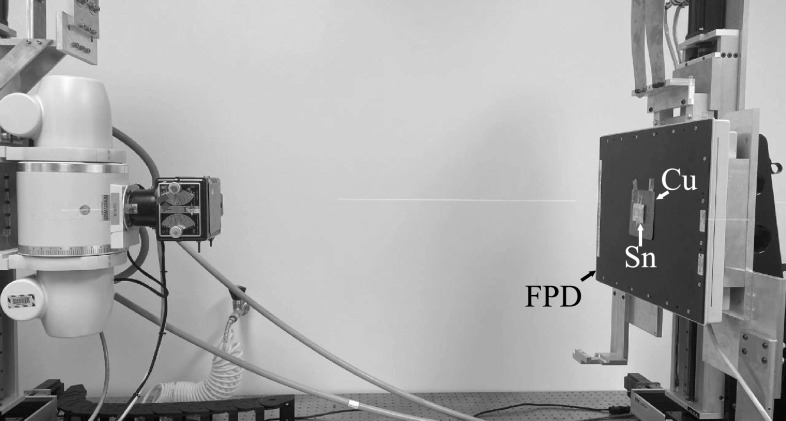
Experimental x-ray imaging benchtop system used in this work.

**Figure 4.**
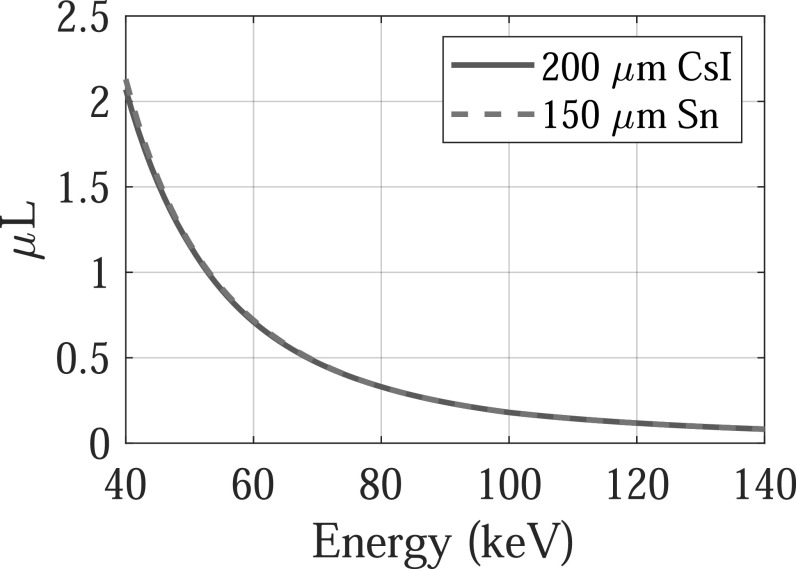
Comparison of attenuation coefficient-thickness product (*μ* *L*) of 150 *μ*m thick tin (Sn) with 200 *μ*m thick cesium iodine (CsI).

No matter whether a DL-FPD uses a permanent or removable filter, the impact of the inter-scintillator filter on the absorption efficiency of the first CsI layer is minimal. Instead, the impact is primarily on the second CsI layer behind the filter. Therefore, the experimental study focused on demonstrating the advantages of the proposed DL-liquid-FPD for the absorption efficiency of the second CsI layer. To do so, an experimental setup shown in figure 3 was used to emulate dual-layer FPDs: a 150 *μ*m thick tin (*Z* = 50) foil was used as a surrogate for the first CsI (*Z*_eff_ = 54) layer: as shown in figure 4, 150 *μ*m tin has very similar x-ray attenuation properties as 200 *μ*m CsI. When the tin (Sn) foil was directly attached to the surface of an FPD with 600 *μ*m CsI, an emulator of the proposed DL-FPD operated under the SE mode was created; when a 1 mm Cu filter was sandwiched between the Sn and the FPD surface, an emulator of the existing DL-FPD was created. The digital FPD (4030CB, Varian Medical Systems, Inc., UT, USA) has a single scintillator (CsI) layer with a thickness of 600 *μ*m, which is similar to the thickness of the 2nd scintillator layer (550 *μ*m) in the existing DL-FPDs. The FPD has 2048 × 1536 pixels with an isotropic pixel pitch of 194 *μ*m. Other key components of the experimental system include a rotating tungsten anode x-ray tube (G1952 with B-180H housing, Varian Medical Systems, Inc., UT, USA) and an 80 kW high-frequency generator (Indico100, CPI Inc., Ontario, Canada).

The zero-frequency noise equivalent quanta (NEQ) of the 600 *μ*m CsI layer was measured at different tube potential levels ranging from 70 to 125 kVp (highest available in our system), all with 2.5 mm inherent filtration and 1 mm Cu added filtration. The exposure per image frame ranges from 8.8 *μ*R (70 kVp) to 126 *μ*R (125 kVp). At each kVp, 250 image frames were repeatedly acquired at 1 mA and 7.5 frames per second. Next, the 1 mm Cu between the Sn layer and the CsI layer on the FPD was removed in order to create a physical emulator of the proposed DL-FPD operated under the SE imaging model. Measurements of the NEQ were repeated under the same input radiation condition.

**Figure 5.**
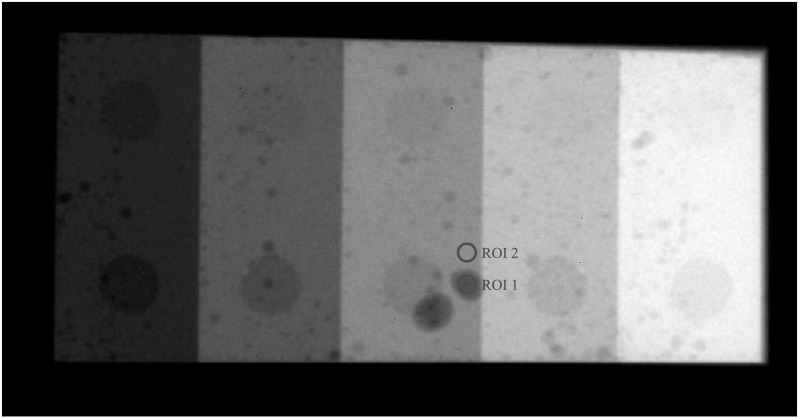
A physical phantom with low-contrast features used in the experimental study. Two circular regions of interest (ROIs) were used to measure the contrast-to-noise ratio (CNR).

In addition to NEQ measurements, a physical phantom with low-contrast features shown in figure 5 was imaged under conditions that simulate (1) existing DL-FPD with a permanent 1 mm Cu filter or (2) proposed DL-FPD (SE mode), respectively. For each condition, an ensemble of 100 image frames was acquired, from which image noise and contrast-to-noise ratios (CNRs) of low-contrast features were measured using the circular ROIs shown in figure 5.

**Figure 6.**
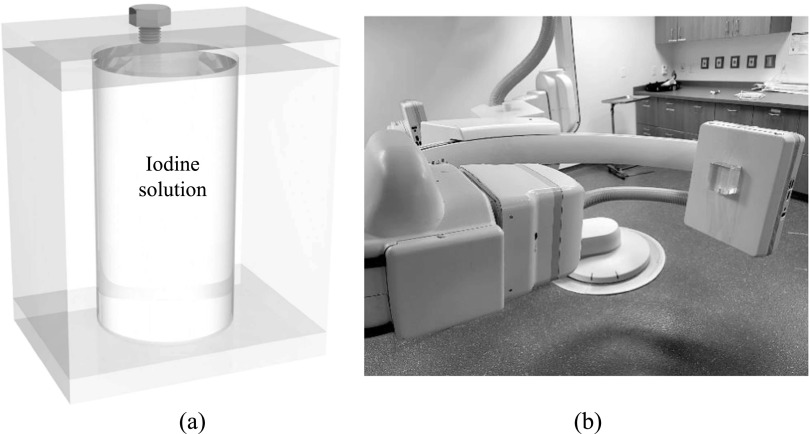
(a) A PMMA phantom with a cylindrical chamber of liquid iodine solution. (b) The phantom was attached to the FPD surface of a C-arm x-ray system to experimentally test the stability of the iodine concentration during gantry rotation.

Finally, to test the stability of liquid filters during gantry rotation, a phantom shown in figure 6 was attached to an FPD of a commercial C-arm x-ray interventional system (Artis Zee, Siemens Healthineer). The phantom contains a cylindrical cavity with a diameter of 5 cm. The cavity was filled with an iodine solution prepared by mixing Omnipaque 350 (GE Healthcare) with deionized water. The iodine concentration is 75 mg cm^−3^, which gives an area density of 375 mg cm^−2^ at the center of the phantom. The C-arm gantry was rotated at its highest speed using a 5 s cone-beam CT scan protocol. X-ray attenuation image of the phantom was recorded at gantry angles ranging from 0 to 200 degrees.

## 3. Results

**Figure 7.**
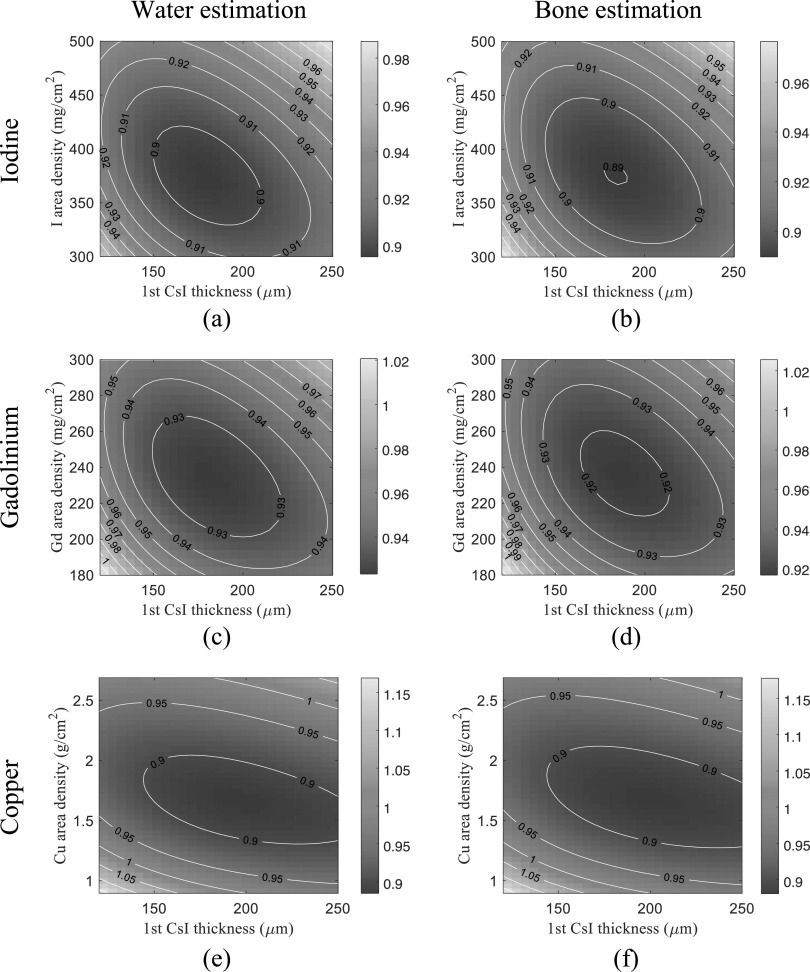
Normalized CRLBs of DL-FPD for water and bone decomposition tasks under the condition of a 120 kVp beam (filtration: 2.5 mm Al+ 1 mm Cu), true *A*_w_ = 30 g cm^−2^, and true *A*_b_ = 10 g cm^−2^. The CRLB values were normalized by the CRLBs of the existing DL-FPD with a 1 mm Cu filter. (a)–(b) Liquid iodine filter. (c)–(d) Liquid gadolinium filter. (e)–(f) Solid copper filter.

Figure 7 plots the CRLBs of water-bone decomposition tasks as functions of *s*_f_ (filter material area density) and *T*_1_ (thickness of first CsI layer). All CRLB values were normalized by those of the existing DL-FPD with 1 mm Cu. For liquid iodine filters, the optimal iodine area density and *T*_1_ are 375 mg cm^−2^ and 180 *μ*m, respectively; for liquid Gd filters, the optimal Gd area density and *T*_1_ are 235 mg cm^−2^ and 180 *μ*m, respectively; for solid Cu filters, the optimal Cu area density and *T*_1_ are 1.66 g cm^−2^ and 195 *μ*m, respectively. Compared with Cu and Gd filters, the iodine filter yielded a slightly lower CRLB.

**Figure 8.**
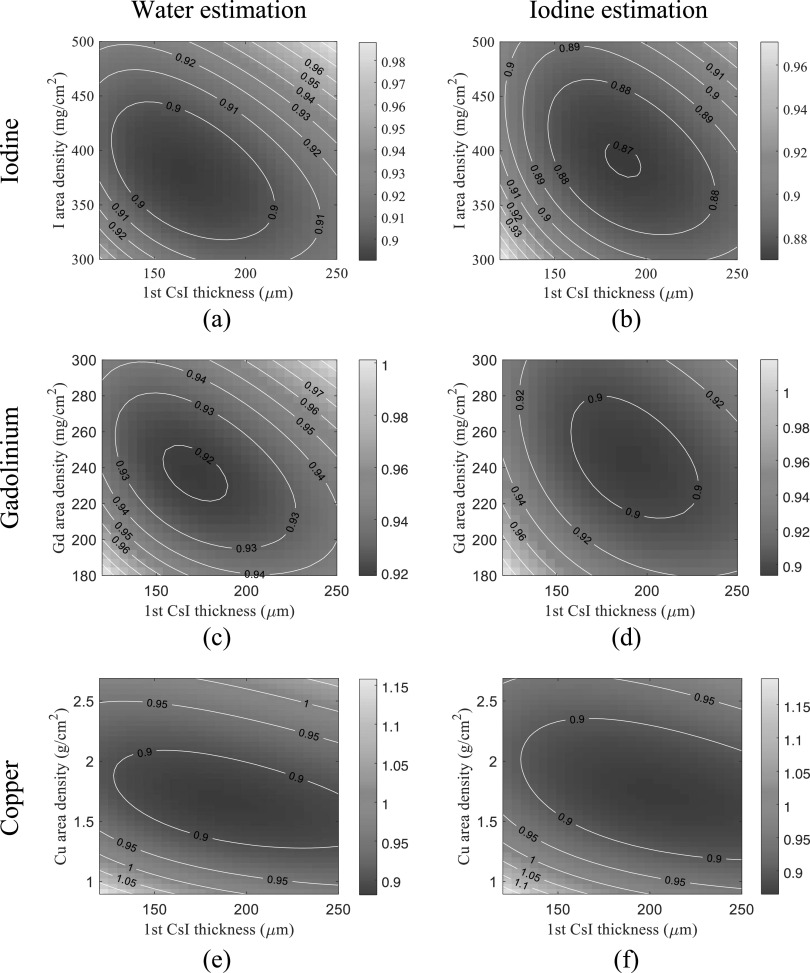
Normalized CRLBs of DL-liquid-FPD for water and iodine decomposition tasks under the condition of a 120 kVp beam (filtration: 2.5 mm Al+ 1 mm Cu), true *A*_w_ = 30 g cm^−2^, and true *A*_I_ = 250 mg cm^−2^. The CRLB values were normalized by the CRLBs of the existing DL-FPD with a 1 mm Cu filter. (a)–(b): liquid iodine filter. (c)–(d): liquid gadolinium filter. (e)–(f): solid copper filter.

Figure 8 plots the normalized CRLBs of water-iodine decomposition tasks as functions of *s*_f_ and *T*_1_. For liquid iodine filters, the optimal iodine area density and *T*_1_ are 380 mg cm^−2^ and 165 *μ*m, respectively; for liquid Gd filters, the optimal Gd area density and *T*_1_ are 235 mg cm^−2^ and 170 *μ*m, respectively; for solid Cu filter, the optimal Cu area density and *T*_1_ are 1.66 g cm^−2^ and 190 *μ*m, respectively. Again, the iodine filter yielded a slightly lower CRLB.

As shown by the contour lines in figures 7 and 8, for a given filter material, a certain deviation from the optimal filter density or *T*_1_ does not substantially change the CRLB values. Therefore, we chose 375 mg cm^−2^ iodine filter and *T*_1_ = 180 *μ*m for the remainder of the theoretical evaluations.

**Figure 9.**
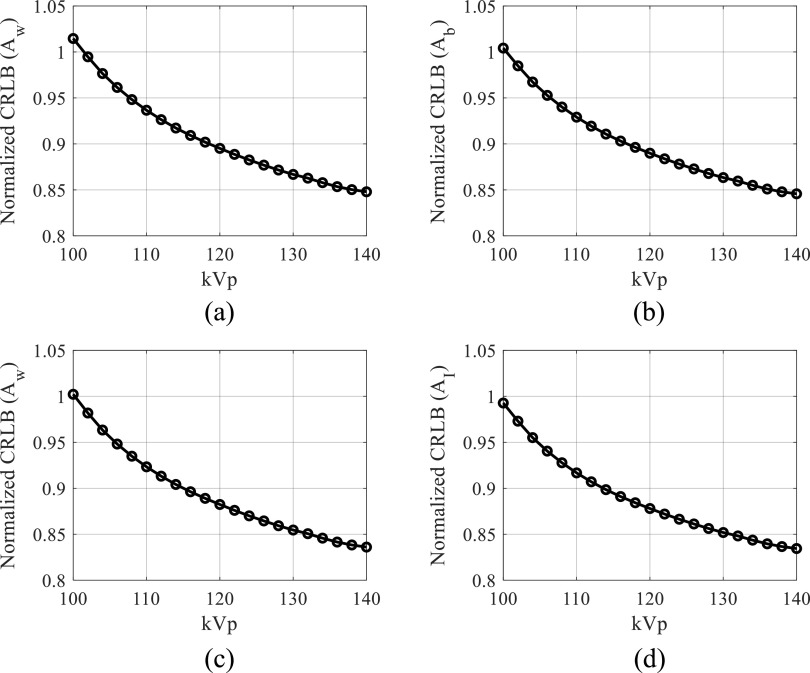
Normalized CRLBs of DL-FPD at different kVp levels. An iodinated liquid filter (375 mg cm^−2^) was used. *T*_1_ = 180 *μ*m; *T*_2_ = 550 *μ*m. (a) Normalized CRLBs of water estimation task in water-bone decomposition. (b) Normalized CRLBs of bone estimation task in water-bone decomposition. In (a) and (b), the true *A*_w_ was assumed to be 30 g cm^−2^; the true *A*_b_ was assumed to be 10 g cm^−2^. (c) Normalized CRLBs of water estimation task in water-iodine decomposition. (b) Normalized CRLBs of iodine estimation task in water-iodine decomposition. In (a) and (b), the true *A*_w_ was assumed to be 30 g cm^−2^; the true *A*_I_ was assumed to be 250 mg cm^−2^.

Figures 9(a)–(b) plot CRLB values of water-bone decomposition DE task as a function of kVp (*A*_w_ = 30 g cm^−2^; *A*_b_ = 10 g cm^−2^). Figures 9(c)–(d) plot CRLB values of water-iodine decomposition DE task as a function of kVp as a function of kVp (*A*_w_ = 30 g cm^−2^; *A*_I_ = 250 mg cm^−2^). All CRLB values were normalized by those of the existing DL-FPD with 1 mm Cu. When kVp is greater than 100, using the iodinated filter (375 mg cm^−2^) improved the DE material decomposition performance when compared with the 1 mm Cu filter.

**Figure 10.**
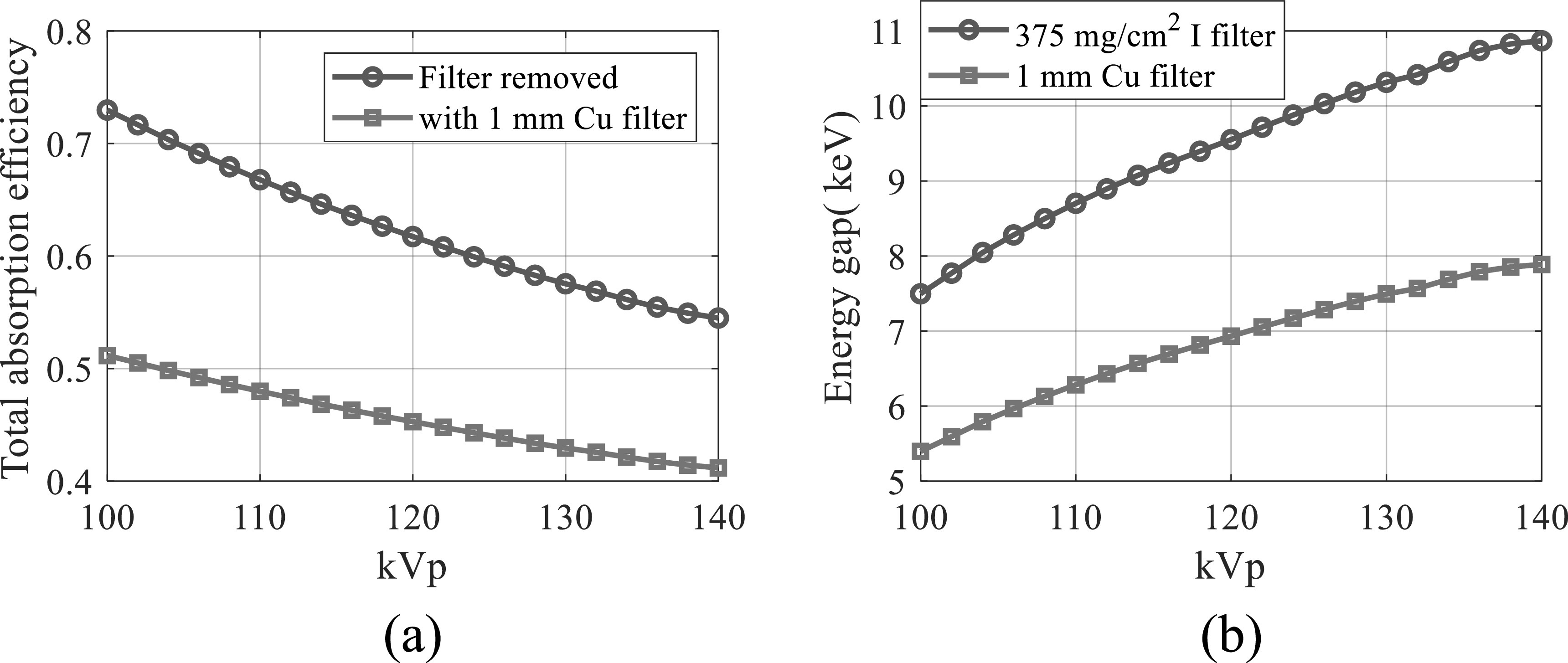
Performance of DL-FPDs at different kVp levels. (a) Total x-ray absorption efficiency. (b) The energy separation between the two scintillator layers. *T*_1_ = 180 *μ*m; *T*_2_ = 550 *μ*m.

Figure 10(a) plots the kVp-dependent of the total x-ray absorption efficiency of the proposed DL-FPD with the filter being removed. As a comparison, the total x-ray absorption efficiencies of the existing DL-FPD with the permanent 1 mm Cu filter are also presented. Thicknesses of the top and bottom scintillators were assumed to be 180 *μ* and 550 *μ*m, respectively. Compared with the existing DL-FPD with the permanent Cu, the relative improvement in the absorption efficiency by the removable filter design ranges from 32% at 140 kVp to 43% at 100 kVp.

Figure 10(b) plots the energy separation between the two scintillator layers in DL-FPD as a function of kVp. When an iodinated filter (375 mg cm^−2^) was used, the energy separation ranges from 7.5 keV at 100 kVp to 10.9 keV at 140 kVp. In contrast, the energy separation in the existing DL-FPD with 1 mm Cu ranges from 5.4 keV at 100 kVp to 7.9 keV at 140 kVp.

**Figure 11.**
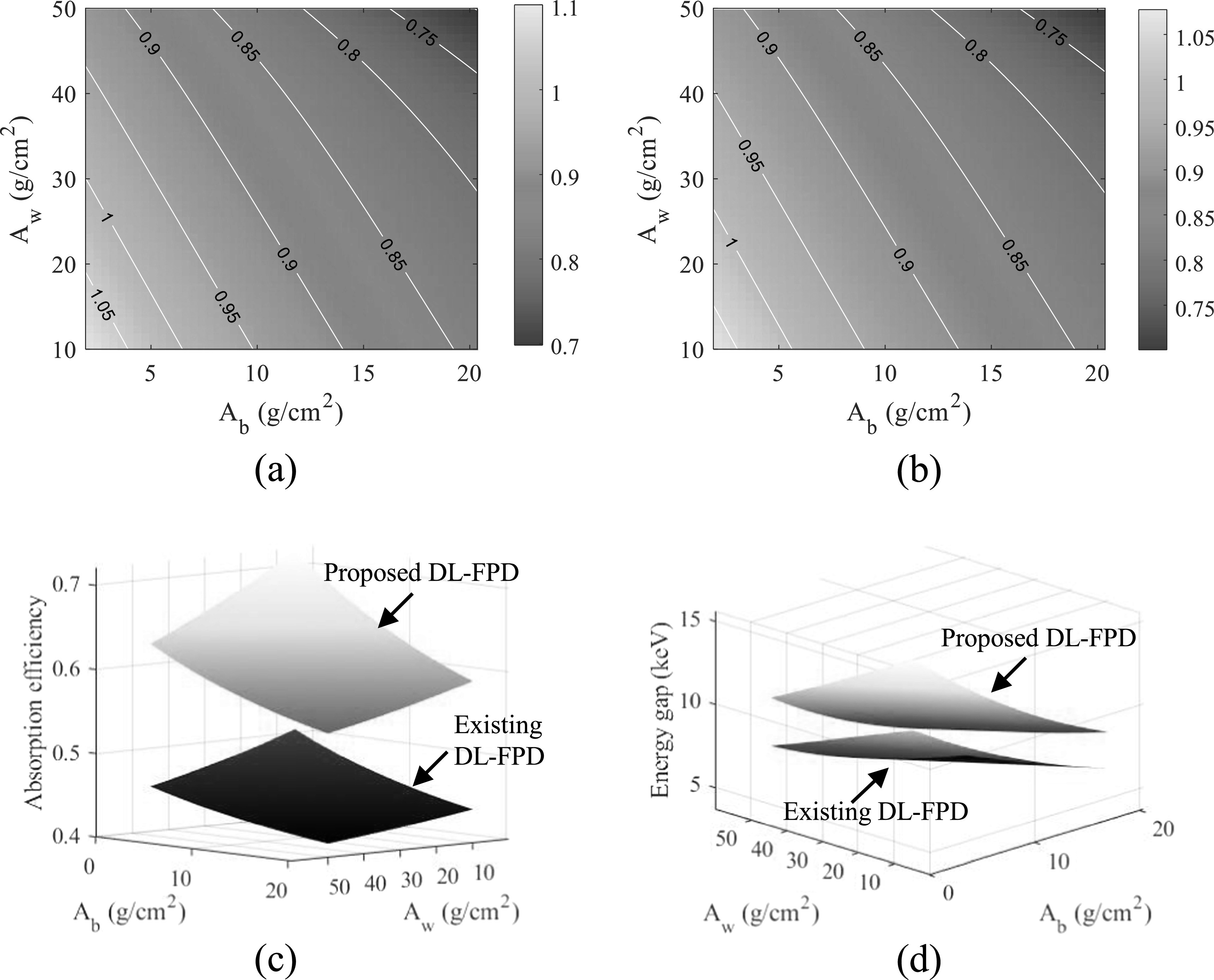
DL-FPD performance at 120 kVp with different object thicknesses. (a) Normalized CRLBs of proposed DL-FPD for the water estimation task plotted as a function of *A*_w_ and *A*_b_. The CRLB values were normalized by the CRLBs of the existing DL-Cu-FPD. (b) Normalized CRLBs of proposed DL-FPD for the bone estimation task. (c) The two scintillator layers’ spectral separation. (d) Total x-ray absorption efficiency. *T*_1_ = 200 *μ*m; *T*_2_ = 550 *μ*m. The proposed DL-FPD used an iodine filter (375 mg cm^−2^).

Using the 375 mg cm^−2^ iodine filter, CRLBs of the proposed DL-FPD were calculated for a variety of *A*_w_ and *A*_b_ values. As shown in figures 11(a)–(b), for the majority of water-bone estimation tasks, the proposed DL-FPD with the optimized iodine filter has a better DE performance, especially at larger *A*_w_ and *A*_b_. As shown by figure 11(c), the proposed DL-FPD with the optimized iodine filter provides a wider spectral separation that improves the DE imaging performance for most tasks. Relative to the exiting DL-FPD with the permanent Cu filter, the proposed DL-FPD improved total x-ray absorption efficiency when the filter was removed from the inter-scintillator space. This improvement is due to the fact that no x-rays are lost in the inter-scintillator filter. The relative improvement in the absorption efficiency depends on the object thickness (*A*_w_ and *A*_b_) and ranges from 32% to 42%.

**Figure 12.**
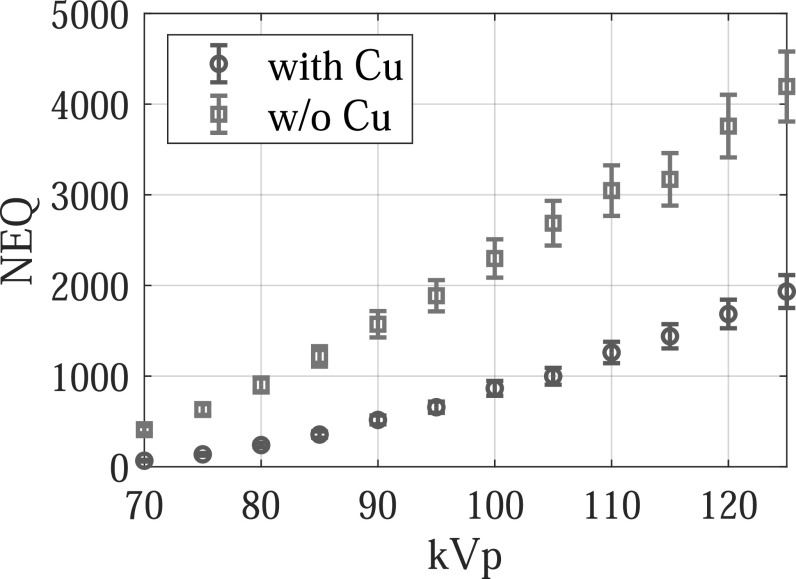
Zero-frequency noise equivalent quanta (NEQ) experimentally measured using an FPD that served as a surrogate for the second CsI layer in dual-layer FPDs.

**Figure 13.**
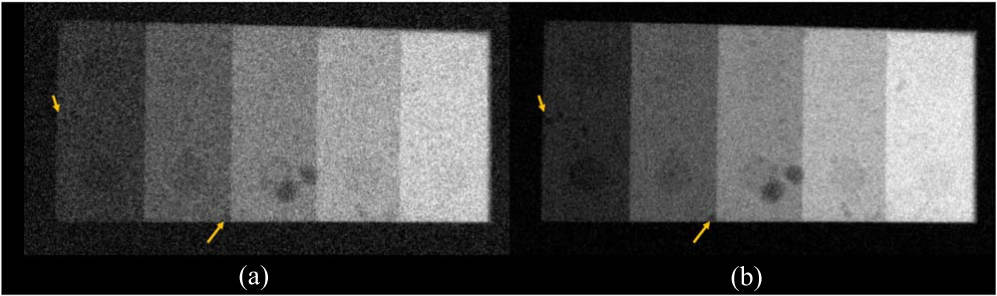
Post-log phantom images acquired using experimental setups that emulate the outputs of the second CsI layer in (a) the DL-Cu-FPD and (b) DL-liquid-FPD with the liquid filter being drained. The arrows point to some low-contrast features in the phantom.

**Figure 14.**
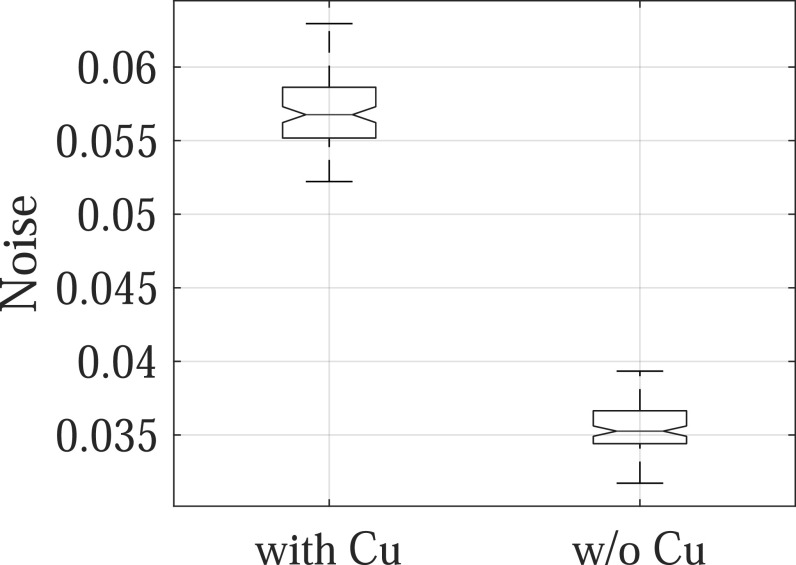
Noise standard deviation of post-log phantom images.

The experimental results are consistent with the theoretical results: as shown in figure 12, removing the Cu filter significantly improved the NEQ level of the FPD that served as a surrogate for the second CsI layer in dual-layer FPDs. For example, at 120 kVp, removing the Cu layer increased the NEQ from 1685 ± 158 to 3758 ± 346, indicating that the Cu layer introduced a loss of 2073 ± 380 in NEQ. Therefore, having a permanent Cu layer is highly undesirable for SE imaging applications. Post-log images of the phantom in figure 13 also confirmed that removing the Cu filter during SE imaging can effectively improve low-contrast detectability: for DL-FPD with a permanent 1 mm Cu filter, the measured CNR is 2.1 ± 0.3; for the proposed DL-FPD, the measured CNR was improved to 3.8 ± 0.3. The CNR improvement is primarily caused by better utilization of the x-rays and a reduction in noise: as shown in figure 14, removing the Cu filter during SE imaging reduces noise standard deviation from 0.057 ± 0.002 to 0.035 ± 0.002.

**Figure 15.**
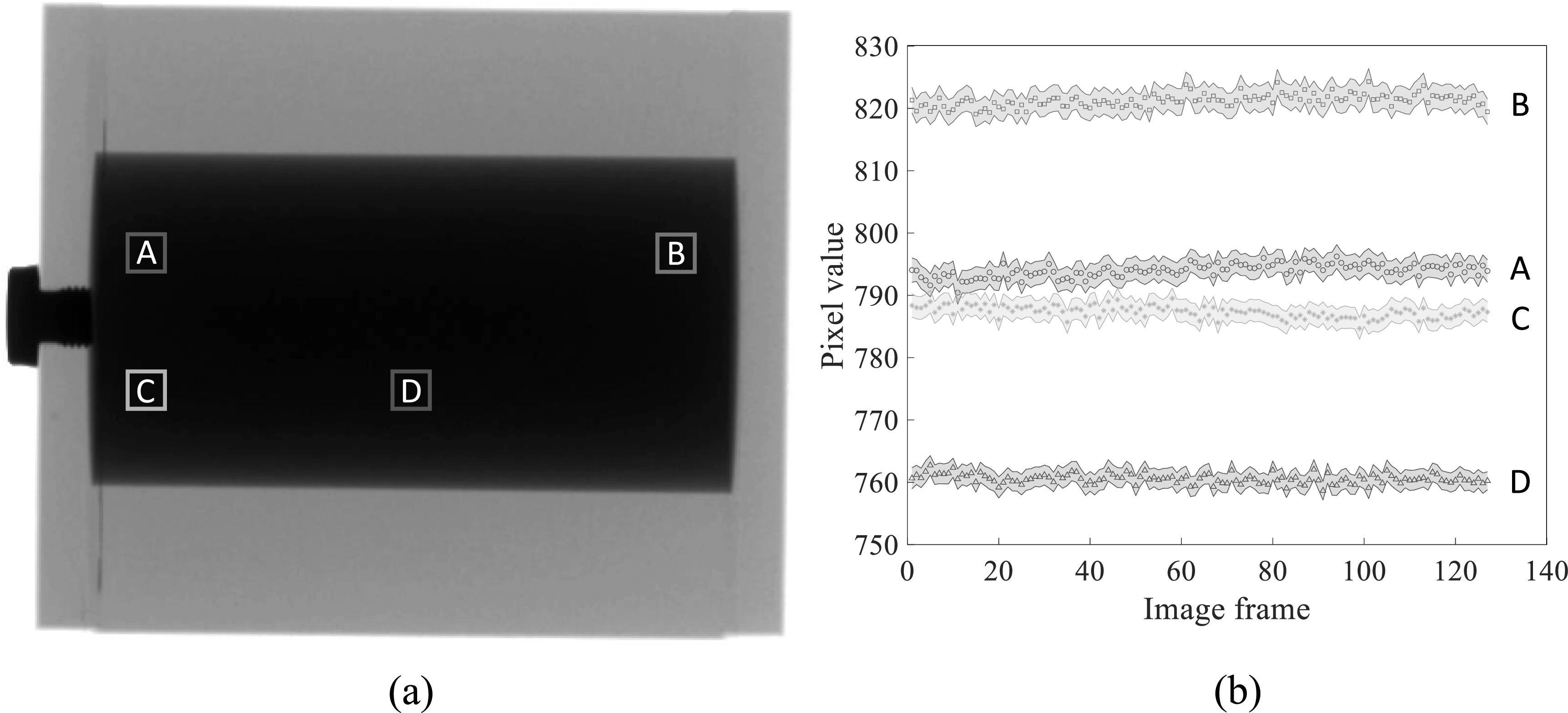
(a) An x-ray image of a liquid iodine phantom. (b) Stability of the liquid iodine solution’s x-ray signal during a C-arm gantry rotation. The shaded areas around the discrete data points represent 95% confidence intervals.

Finally, figure 15 shows that for each local region, the attenuation signal of the liquid iodine is very stable over the course of C-arm gantry rotation at its highest speed (5 s/scan), despite the presence of centrifugal and gravitational forces and possible gantry wobbling. Because of this stability, we expect that a flood-field normalization will cancel out the liquid iodine signal when it is used as a filter in DL-FPD’s DE imaging procedures.

## 4. Discussion

Compared with the dual-layer detector design reported in the 1986 paper by Barnes *et al* (1985), the more recent DL-FPD prototypes have a much larger *T*_1_ (thickness of 1st scintillator layer) and a much thicker Cu layer. Although the precise reason for making these changes is unknown, increasing *T*_1_ does help improve the overall x-ray absorption efficiency: based on the Beer–Lambert law, a thicker 1st scintillator layer reduces the x-ray intensity reaching the front surface of the Cu layer, and thus the total amount of x-ray loss in Cu can be reduced. However, a thicker 1st scintillator layer also absorbs more high-energy x-rays, which pushes the mean energy of the absorbed spectrum closer to that of the 2nd scintillator layer. In that case, using a thicker (i.e. 1 mm) Cu filter imposes a more aggressive beam hardening to the penetrated x-rays to maintain a decent spectral separation between the two scintillator layers. Despite this, the spectral separation of the current DL-FPD is still narrower compared with the one in Barnes *et al* (1985). An important advantage of the proposed DL-FPD is that, when optimizing the scintillator and filter thicknesses, no additional consideration of the SE imaging performance is needed due to the removable filter design. In other words, the design is no longer restricted by the trade-off between total absorption efficiency and energy separation. This allows for an optimal design of the dual-layer FPD for DE imaging.

As illustrated in figure 2, the removable filter can be implemented using either a liquid solution or a solid material. Using liquid filters gives the potential of automated filter engagement and removable. This is particularly attractive for robotic C-arm systems for interventional applications, during which SE and DE imaging modes often need to be switched back and forth and the whole FPD can be wrapped by a protective bag. Solid filters usually have higher mechanical stability and can be inserted or removed manually. Therefore, no additional pumps or purging devices are needed as in the case of liquid filters. Solid filters can be potentially implemented using resin plates with iodine/gadolinium additives of the desired concentration. Alternatively, solid filters can be implemented using metallic materials. Since iodine or Gd solutions outperformed Cu as shown in figures 7 and 8, the DE imaging performance of the DL-FPD should benefit from the use of a high-atomic number material. Doing an exhaustive search of the metallic filter materials is needed in the future but is out of the scope of this paper, which focuses on the presentation of the removable filter concept.

For liquid materials, the front and back walls of the filter chamber need to be made of thin and low-attenuating materials to reduce x-ray loss. For example, we experimentally measured the impact of two 1/16″ (1.6 mm) acrylic sheets on x-ray intensity under the condition of 120 kVp, 1 mm Cu external beam filtration, and 150 *μ*m Sn (to emulate the beam hardening by the first CsI layer). The percent reduction in the measured signals of our FPD is only 3.6% ± 0.6%, indicating a negligible impact from the acrylic sheets on x-rays.

Compared with solid metallic filters, liquid filters with PMMA chamber walls have lower thermal conductivities. However, the passive a-Si TFT panel generally has a very low power consumption. Therefore, no heat dissipation problem is anticipated when assembling a TFT panel directly with a liquid filter chamber. This is evidenced by the commercial availability of triple-layer FPDs, in which the top CsI/TFT unit is followed by a middle CsI/TFT unit and then by a third (bottom) CsI/TFT unit (Karim 2021).

In practice, to help avoid the problem of residual liquid on the chamber during the SE imaging mode, special treatments of the chamber wall material may be needed. There exist methods to make the walls superhydrophobic and thus have the liquid completely bead off in the SE operating mode, whether they involve a coating, a treatment, or something mixed into the wall material itself. One method is to coat the walls with SiO_2_ or TiO_2_ using a sol-gel process (Huang *et al* 2017); another is to use polydimethylsiloxane (PDMS)-PMMA combination as a spray coating (Liu *et al* 2017) or a polystyrene-PMMA combination as a spin coating (Ma *et al* 2007). It is also possible to use a low-temperature plasma to treat and change the hydrophobicity properties of the surface of the material (Xu *et al* 2013).

When an image object is projected onto a DL-FPD system, its geometric magnification is different between the two scintillator layers. Therefore, the impact of filter thickness on the geometric mismatch needs to be considered when building the proposed detector. Prior works have demonstrated that, by incorporating system optics models into image processing and reconstruction, the geometric mismatch can be effectively compensated even for high-resolution imaging applications (Wang *et al* 2020, 2021).

DL-FPDs discussed in this work may find applications in x-ray digital radiography systems, mobile C-arm x-ray systems, and C-arm systems for image-guided interventions and radiotherapy. The proposed DL-FPDs were not designed for use in multi-detector row CTs (MDCTs), which have much faster gantry rotation speeds compared with C-arm CBCTs. A fast-rotating MDCT gantry can exert a strong centrifugal force on the liquid filter material and introduce instabilities to the filter’s x-ray absorption.

This work has the following limitations: First, the detector optimization only studied three filter materials (I, Gd, and Cu) and two general groups of DE imaging tasks (water and bone thickness estimations via water-bone 2-material decomposition; water and iodine thickness estimations via water-iodine 2-material decomposition). Further, the scope of this paper is limited to single-layer removal filters, i.e. we only studied one material at a time for the proposed removable filter. It is in principle possible to combine multiple solid filters of different materials and thicknesses, or use a different material, other than acrylic, to build the chamber for holding the liquid. Whether multi-layer removable filters can bring additional benefits to certain imaging tasks deserves investigation in the future. Next, the work only studies one scintillator material (CsI, *Z* = 54). In the 1986 paper by Barnes *et al* (1985), Y_2_O_2_S (*Z* = 35) was used in the 1st scintillator layer while Gd_2_O_2_S (*Z*_eff_ = 58) was used in the 2nd scintillator layer. The difference in *Z* between Y_2_O_2_S led to a larger spectral separation (14.8-23.6 keV with 140 kVp inputs) (Barnes *et al* 1985) compared with the more recent CsI DL-FPDs (Lu *et al* 2019). Although the majority of medical FPDs use CsI due to its better spatial resolution properties by growing CsI into columnar structures (Miller *et al* 2005), it is interesting to explore in future works the joint use of Y_2_O_2_S (as the first scintillator layer) and the proposed liquid filter device for building DL-FPDs. Third, the scope of this work does not include the actual fabrication of the DL-FPD device. Our focus was put on the theoretical optimization of the liquid filter material, which did not consider the impacts of x-ray scattering, scintillation light loss and spatial spreading, electronic noise of the readout systems, additional x-ray attenuation by the TFT panels, walls of the liquid filter device, and foam protective layers in front of the scintillator. For material decomposition and other DE imaging tasks, hardware- or software-based scatter correction is often applied, which mitigates the influence of scattered x-rays but also changes the spectral and statistical models of *I*_1_ and *I*_2_. The most straightforward approach to include scattering and other factors in the evaluation of the proposed DL-FPD is to physically build such a device. This will be an important task for our future work.

## 5. Conclusions

For existing DL-FPDs with a permanent Cu filter layer, over 30% of the input x-rays can be lost in the Cu layer regardless of the use of DE or SE imaging modes. In this work, a novel dual-layer FPD design was proposed and optimized. By making the inter-scintillator filter removable using either liquid or solid materials, the DL-FPD can be toggled between DE imaging mode and high-absorption-efficiency SE imaging mode. The removable design also decouples the spectral separation consideration from the x-ray absorption efficiency consideration when designing the DL-FPD, which permits improvements in DE imaging performance as well.

## Data Availability

The data cannot be made publicly available upon publication because they are not available in a format that is sufficiently accessible or reusable by other researchers. The data that support the findings of this study are available upon reasonable request from the authors.
